# Australian Wild Rice Reveals Pre-Domestication Origin of Polymorphism Deserts in Rice Genome

**DOI:** 10.1371/journal.pone.0098843

**Published:** 2014-06-06

**Authors:** Gopala Krishnan S., Daniel L. E. Waters, Robert J. Henry

**Affiliations:** 1 Southern Cross Plant Science, Southern Cross University, Lismore, New South Wales, Australia; 2 Division of Genetics, Indian Agricultural Research Institute, New Delhi, India; 3 Queensland Alliance for Agriculture and Food Innovation, The University of Queensland, Brisbane, Queensland, Australia; University of Florence, Italy

## Abstract

**Background:**

Rice is a major source of human food with a predominantly Asian production base. Domestication involved selection of traits that are desirable for agriculture and to human consumers. Wild relatives of crop plants are a source of useful variation which is of immense value for crop improvement. Australian wild rices have been isolated from the impacts of domestication in Asia and represents a source of novel diversity for global rice improvement. *Oryza rufipogon* is a perennial wild progenitor of cultivated rice. *Oryza meridionalis* is a related annual species in Australia.

**Results:**

We have examined the sequence of the genomes of AA genome wild rices from Australia that are close relatives of cultivated rice through whole genome re-sequencing. Assembly of the resequencing data to the *O. sativa* ssp. *japonica* cv. Nipponbare shows that Australian wild rices possess 2.5 times more single nucleotide polymorphisms than in the Asian wild rice and cultivated *O. sativa* ssp. *indica*. Analysis of the genome of domesticated rice reveals regions of low diversity that show very little variation (polymorphism deserts). Both the perennial and annual wild rice from Australia show a high degree of conservation of sequence with that found in cultivated rice in the same 4.58Mbp region on chromosome 5, which suggests that some of the ‘polymorphism deserts’ in this and other parts of the rice genome may have originated prior to domestication due to natural selection.

**Conclusions:**

Analysis of genes in the ‘polymorphism deserts’ indicates that this selection may have been due to biotic or abiotic stress in the environment of early rice relatives. Despite having closely related sequences in these genome regions, the Australian wild populations represent an invaluable source of diversity supporting rice food security.

## Introduction

Food security depends on sustainable crop production; especially for the major cereals such as rice, which contributes to more than half of the human food across the globe. Enhancing rice production to meet growing food demand requires continuous genetic improvement, especially in response to biotic and abiotic stress that may be intensified by climate change. The wild gene pool of Asian cultivated rice (*Oryza sativa*) is found in Asia and Australia [1]. Much of the Asian wild rice populations have been displaced by cultivated rice since domestication started in China around 7000 years ago [2]. Gene flow from large domesticated Asian rice populations has also impacted the small remaining populations of Asian wild rice.

As in other domesticated plants, the gene pool of cultivated rice has reduced genetic diversity relative to that found in the wild due to the bottleneck imposed by domestication, harbouring genomic regions which are significantly depressed in diversity [3],[4]. Australian wild rice populations have been isolated from domesticated rice and are a reservoir of environmentally adapted genetic diversity which could be exploited for cultivated rice improvement [5].

In the present study, we have undertaken whole shotgun genome sequencing of wild Australian and Asian rices and aligned the sequences with the cultivated rice, *O. sativa* ssp. *japonica* cv. Nipponbare reference genome. Based on the analysis of the variations in DNA polymorphisms across the entire rice genome, we were able to identify ‘polymorphism deserts’ in the genomic regions possessing genes for adaptive traits. Our analysis shows that this reduction in polymorphism is not restricted to cultivated *Oryza* species but also found in the wild species of *Oryza* from Australia also, suggesting the role of predomestication bottleneck induced due to natural selection.

## Materials and Methods

### Germplasm, sampling and sequencing

The *Oryza sativa* ssp. *indica* germplasm is an elite parental line used in hybrid breeding programme. The Asian *O. rufipogon* strain was from the Australian Plant DNA Bank Number (AC11-1008369). The sample was collected by Ryuji Ishikawa in a collaboration approved by Nguyen Thi Lang, Head of Genetics and Plant Breeding Division, Cuulong Delta Rice Research Institute, Can Tho, Vietnam, from a site located at N9 59.376 E105 39.883. This species is not endangered. The field studies did not involve endangered or protected species. Australian *O. rufipogon* was sourced from the Australian Tropical Crops and Forages Collection, Biloela (AusTRCF 309313; Australian Plant DNA Bank Number - AC01-1002323; collected from site located at N 18.206, E 142.865, about 0.9 K west Gilbert River Bridge in Gulf Development Road). *O. meridionalis* was sourced from the Australian Tropical Crops and Forages Collection, Biloela (AusTRCF 300118_B; originally collected Northern Territory, Australia). Construction of library and sequencing of these germplasm accessions was performed on Illumina Genome Analyser (GAIIx) with detailed procedure as described previously [6]. Paired end reads generated from all the genotypes were deposited in the NCBI sequence read archive (SRA) and can be found under the accession number SRP039365 (*Oryza sativa* ssp. *indica* - SRX480815; Asian *O. rufipogon* - SRX480820; Australian *O. rufipogon* - SRX480822 and *O. meridionalis* - SRX480817).

### Mapping reads to the reference and SNP calling

Paired-end sequence reads were trimmed of low-quality data with a quality score limit of 0.01 and adaptor sequence in CLC Genomics Workbench 4.0 (http://www.clcbio.com) and reads of less than 30 base pairs (bp) in length were discarded. Trimmed short-read sequences were first aligned to the published rice organellar genomes (Chloroplast genome: Genbank accession - AY522330.1, mitochondrial genome: Genbank accession - DQ 167400.1) and the unmapped reads were taken up for further assembly against the nuclear genome (IRGSP Pseudomolecules build 4.0, http://rgp.dna.affrc.go.jp/IRGSP/Build4/build4.html). The reads were assembled to the Nipponbare reference with CLC Genomics workbench with the following parameters: mismatch cost - 2, insertion cost - 3, deletion cost - 3, length fraction - 0.5 and similarity - 0.8. Reads that aligned to more than one position of the reference genome were filtered and only unique reads were used for calling the SNPs. For comparison with 93–11 genome, the reads were first aligned to organellar genomes (Chloroplast genome: Genbank accession - AY522329.1, mitochondrial genome: Genbank accession - DQ167399.1) and the unmapped reads were aligned against the nuclear genome (Genbank accession - AAAA02000000) using the above parameters. SNPs in the assembled contigs relative to the reference genome were identified with detailed procedure as described previously [6].

### Analysis of variations

To quantify the DNA polymorphisms across the genome in different species, a sliding window of 100 kb intervals was used to analyse each chromosome to determine SNP frequency in each window.

### Analysis of ‘Polymorphism desert’

Comparison of the mean SNP frequency within the region equivalent to the chromosome 5 ‘polymorphism desert’ of cultivated rice (8.97 and 13.56 Mbp) and the remainder of chromosome 5 was by t-test. The analysis of coverage and SNPs within the ‘polymorphism desert’ was carried out as described in File S1. Annotated genes within the ‘polymorphism desert’ equivalent region were retrieved from the IRGSP Pseudomolecules Build 4. RAP-DB ID Converter (http://rapdb.dna.affrc.go.jp/tools/converter) was used for converting the Locus ID of each gene to the corresponding MSU locus identifier. The functions of the genes were retrieved from the Rice Genome Annotation Project website (http://rice.plantbiology.msu.edu).

### Analysis of selection sweeps

The SNP variations in 2 Mb region centred around 15 cloned genes in rice namely *Gn1a* [7], *Rd* [8], *qSH1* [9], *sd1* [10] – [12], *GW2* [13], *GS3* [14], *GIF1* [15], *Bh4* [16], *sh4* [17], *qSW5* [18], *wx* [19], *PROG1* [20], *Rc* [21], *GBSSII* [22] and *BAD2* [23] which has undergone selection either during domestication or crop improvement was assessed using a sliding window of 1kb interval and the selective sweeps were determined as reflected by a significant reduction in mean SNPs/kb (Text S1).

## Results and Discussion

Whole genome re-sequencing yielded 51859475, 67186809, 62321354 and 46409181 paired reads of raw data in *Oryza sativa* ssp. *indica*, Asian *O. rufipogon*, Australian *O. rufipogon* and *O. meridionalis*, respectively. The reads were 75-bp paired end in case of reads *Oryza sativa* ssp. *indica*, while in case of Asian *O. rufipogon*, Australian *O. rufipogon* and *O. meridionalis*, it was 36-bp paired end reads. After appropriate processing, the short reads were mapped to high quality genomic sequences of *japonica* rice cultivar, Nipponbare using CLC Genome workbench 4.0. A total of 4773330, 6231758, 1013433 and 1644640 reads mapped to the organellar genomes; and 32723087, 32060326, 43606315 and 25523774 reads from *Oryza sativa* ssp. *indica*, Asian *O. rufipogon*, Australian *O. rufipogon* and *O. meridionalis*, respectively were uniquely mapped to the 12 pseudomolecules of the Nipponbare genome. On an average, the sequencing depth of 5.7X, 8.4X, 6.2× and 4.9× across the whole genome, providing genome coverage of 76.2%, 78.2%, 63.2% and 62.6% of the Nipponbare reference genome in case of *Oryza sativa* ssp. *indica*, Asian *O. rufipogon*, Australian *O. rufipogon* and *O*. *meridionalis*, respectively.

The analysis of genome-wide polymorphisms revealed that the number of SNPs detected in cultivated *O. sativa* ssp. *indica* was only 978,630 as compared to 2,564,013 SNPs in Australian *O. rufipogon* relative to *O. sativa* ssp. *japonica* cv. Nipponbare reference genome (Table S1). There were in the order of 2.5 times more single nucleotide polymorphisms (SNPs) in the Australian wild rice than in the Asian wild rice and cultivated *O. sativa* ssp. *indica* relative to *O. sativa* ssp. *japonica* cv. Nipponbare (Table 1), highlighting the potential value of the Australian wild populations as sources of novel variation for rice improvement. The mean SNPs per kb of genome was 6.7 and 6.3 in Australian *O. rufipogon* and *O. meridionalis*, respectively compared to 2.4 in *O. rufipogon* from Asia and 2.5 in *O. sativa* ssp. *indica*. The mean SNPs per kb of genes observed was 8.06 and 7.84 in Australian wild rice, O. rufipogon and O. meridionalis, respectively compared to 2.13 in O. rufipogon from Asia and 2.12 in cultivated rice (Table S2). The mean number of nonsynonymous SNPs per kb of gene was also up to 3 fold higher in the Australian A genome wild rice (Table S3). *O. rufipogon*, collected from the Mekong delta of Vietnam had fewer SNPs relative to Nipponbare than cultivated *O. sativa* ssp. *indica*, consistent with the hypothesis that *O. sativa* ssp. *japonica* was domesticated directly from *O. rufipogon* and perhaps reflecting the history of pollen flow from cultivated to wild populations [24]. The Mekong delta is the principal rice growing region of Vietnam and it is likely there has been gene flow between the wild *O. rufipogon* and *O. sativa* ssp. *japonica* cultivated in this and many other Asian regions.

**Table 1:** Single nucleotide polymorphisms in cultivated and wild *Oryza* as compared with *O. sativa* ssp. *japonica* cv. Nipponbare.

*Oryza* species	Whole genome		Chromosome 5		Chromosome 5 low diversity region (8.972 -13.557 Mb)	
	Total	SNPs/kb	Total	SNPs/kb	Total	SNPs/kb
*O. sativa* ssp.*indica*	978,630	2.56	68,443	2.28*	244	0.053*
*O. rufipogon* (Asian)	917,738	2.40	70,367	2.34*	6,050	1.302*
*O. rufipogon* (Australian)	2,564,013	6.71	219,794	6.71*	18,179	3.96*
*O. meridionalis*	2,418,084	6.33	206,884	6.89*	17,139	3.73*

* Means significantly different (t–test, p<0.01).

**Figure 1:**
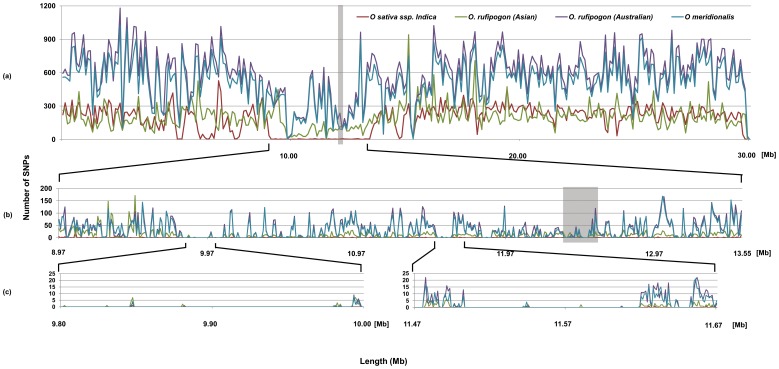
SNP distribution across Chromosome 5. Numbers on vertical axis are SNP/kb. Numbers on vertical horizontal axis are Mb from origin. Vertical grey bar represents the centromere.

Genome wide comparisons between *O. sativa* ssp. *japonica* and *O. sativa* ssp. *indica* cultivars have revealed a low diversity region also referred to as ‘polymorphism desert’ between 8.97 and 13.56 Mbp on chromosome 5 with less than 10 SNP per 100 kb while the mean SNP rate of Chromosome 5 is comparable to the mean SNP rate across other chromosomes [6]. A similar SNP distribution pattern has been observed in *indica-japonica* [25]–[27], *indica-indica* [6], and *japonica*-*japonica* [28] comparisons. Analysis of the equivalent region in Asian *O. rufipogon* and the Australian AA genome wild rice found divergence from cultivated rice to be reduced by more than 40% in these species relative to the chromosome as a whole. This was observed not only based upon comparisons with *O. sativa* ssp. *japonica* cv. Nipponbare (Table 1 and Figure 1) but also with the *O. sativa* ssp. *indica* (cv. 93-11) reference genome sequences (Figure S1).

The chromosome 5 ‘polymorphism desert’ is in the vicinity of the centromere and it has been observed that recombination in centromeric regions is depressed relative to other chromosomal regions which may influence SNP and InDel frequency [25], [29]. In *O. sativa* ssp. *indica*, analysis of SNP distribution within two Mb centred on the centromere of chromosome 5 showed that the mean SNP rate in this ‘polymorphism desert’ was only 0.07 per kb which is very low compared to the chromosomal mean of 2.28 SNPs per kb. Comparatively, chromosomes 4 and 8 had an average SNP rate of 1.79 and 1.33, per kb respectively around the centromere, as compared to the mean SNP rate of 2.21 and 2.48 per kb across these chromosomes. This analysis suggests that the presence of the centromere alone is not likely to explain the ‘polymorphism desert’ on chromosome 5. Additionally, the aligned data was subjected to an analysis which showed that the coverage across chromosome 5 was more than 4 reads ruling out the fact that the ‘polymorphism desert’ in chromosome 5 is not due to lower coverage (Figure S2 and S3). Further, the analysis of SNPs distribution including SNPs from repetitive sequences in chromosome 5 revealed that the reduction in SNPs is not due to filtering of SNPs from repetitive region (Figure S4).

Rice has been cultivated for seven to eight thousand years [2]. During the process of domestication, a range of favourable alleles have been captured [30]. Each time this has occurred, the rice genome has gone through a bottle neck, the remnants of which are most apparent as selective sweeps surrounding genes which code for traits that support rice cultivation [31], [32]. A number of these selective sweeps have been investigated and they have been found to be in the range from 0.3 to 1.0Mb [33]. Within the germplasm studied here, selective sweeps ranging from 151 to 488 kb were apparent around seven of 15 candidate domestication/improvement genes (Table 2, Figure S5) while such signatures of domestication were not detected for another eight domestication related genes examined (Table S4, Figures S6 and S7). In contrast, the chromosome 5 ‘polymorphism desert’ in the cultivated rice is significantly larger in extent in comparison to regions of depressed diversity around known domestication genes. The scale of the ‘polymorphism desert’, 4.58 Mb, suggests this region of the genome may harbour a cluster of several genes important to plant function and perhaps cultivation with overlapping selective sweeps.

**Table 2:** Extent of loss in variation in the domestication and plant improvement genes as reflected by SNP distribution (SNPs per kb) in the 2 Mb genomic region surrounding them.

Locus	Length of low polymorphism region (kb)	*O. sativa* ssp. *indica*	*O. rufipogon* (Asian)	*O. rufipogon* (Australian)	*O. meridionalis*
*GS3*	357	0.07 (25)	2.48 (884)	8.15 (2,910)	7.42 (2,650)
*Bh4*	374	0.26 (98)	3.01 (1,126)	8.86 (3,313)	7.32 (2,737)
*sh4*	371	0.48 (178)	1.87 (694)	7.29 (2,711)	8.73 (3,247)
*qSW5*	151	0.05 (8)	3.46 (522)	10.36 (1,564)	9.61 (1,451)
*wx*	369	0.07 (27)	2.62 (967)	8.7 (3,209)	8.40 (3,098)
*PROG1*	267	0.09 (25)	2.42 (647)	6.12 (1,634)	6.17 (1,647)
*Rc*	488	0.15 (74)	2.78 (1,359)	5.99 (2,922)	4.24 (2,069)

*Numbers in parenthesis is the total number of SNPs detected in the respective regions in comparison with Nipponbare genome.

Given the low level of polymorphism in the ‘polymorphism desert’ within *Oryza sativa* sub-species, approaches which rely on analysis of genetic difference would have difficulty in detecting and identifying genes of significance within this region. Reference to the paralogues in the Australian wild AA genome species which have not been in contact with cultivated rice provides clues as to which genes may have been under selection. Of 143 genes annotated within this region, 93 genes had sufficient coverage (atleast 4 reads) across the genomes sequenced to allow comparison. The Australian wild rices had a significantly higher number of SNPs and non-synonymous SNPs (nsSNPs) in the genes compared to cultivated rice (Tables S2 and S3). However, it was observed that the Australian wild rices also had a low number of SNPs in 61 genes. Gene annotations in the rice genome suggest 16 of these genes are involved in signalling, inflorescence and seed development, Fe and P interaction, disease resistance and seed germination (Table S5). The role of the genes within this region in various functions such as aerobic germination, cytokinin response in roots, Fe and P interaction suggests that these genes might have been subjected to natural selection in the wild rice progenitors prior to domestication; and human selection has magnified the effect in cultivated rice.

### Conclusions

**Figure 2:**
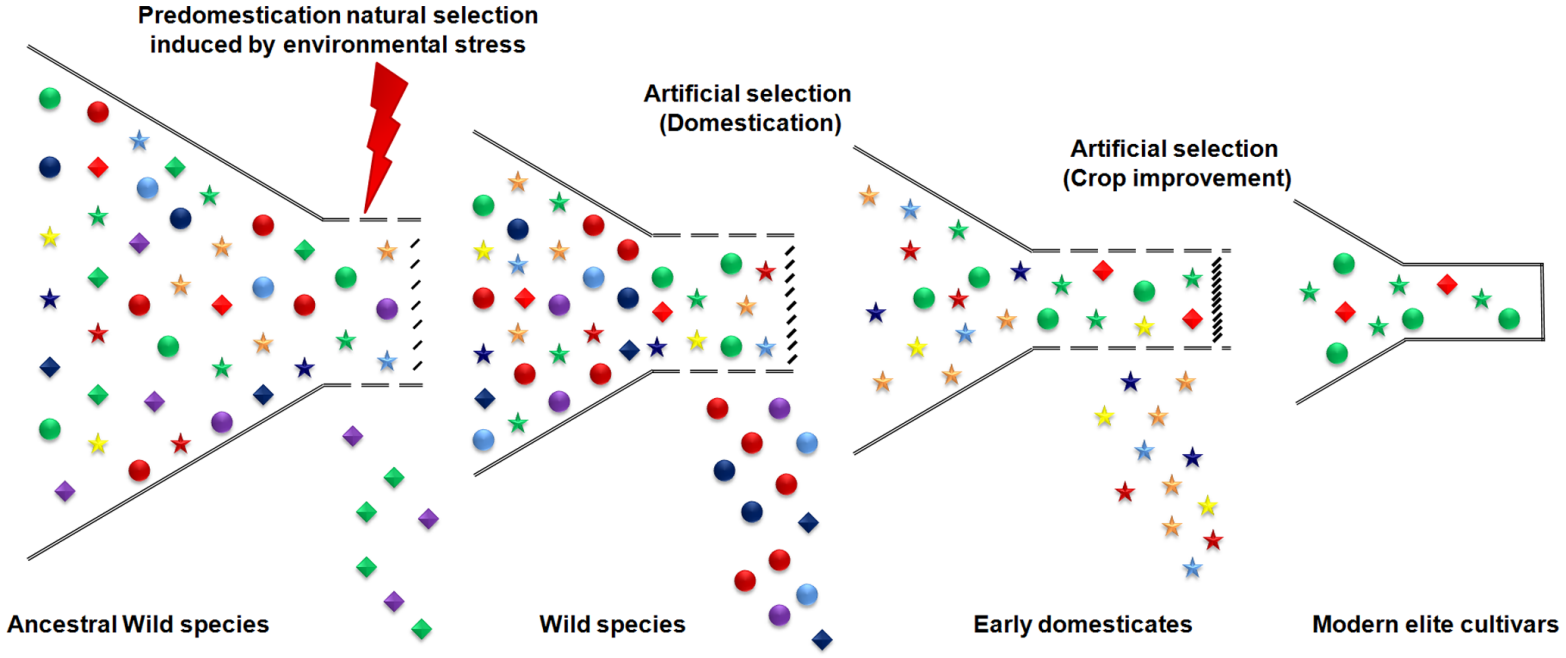
Predomestication bottleneck prior to domestication and crop improvement resulting in ‘Polymorphism deserts’ in cultivated rice. Different classes of genes are represented by different shapes; dice shape (♦) indicates genes for adaptive traits, circles (•) indicates domestication genes, star shape (*) indicates genes for crop improvement. The allelic forms of genes are represented in different colors. A predomestication bottleneck possibly induced by environmental stress resulted in loss of polymorphisms in the adaptive genes in case of wild rice. Additional selection pressure during domestication and crop improvement resulted in further depauperating the polymorphisms resulting in the ‘polymorphism deserts’ as in the case of chromosome 5 of rice. Artificial selection during rice domestication in genes such as *sh4, PROG1* resulted in reduced diversity in the adjoining genomic regions due to selection sweeps associated with the genes. Further selection during crop improvement in genes such as *GS3*, *Bh4*, *qSW5*, *wx* and *Rc* also reduced the polymorphisms in the regions associated with these genes. While the selections during domestication and crop improvement helped in retaining favourable alleles at these loci, an additional pre-domestication bottleneck has resulted in loss of variation in the genes providing adaptive traits in the ‘polymorphism desert’ of Chromosome 5.

The present study shows that the Asian cultivated rice has lost variability as a result of selection during domestication and crop improvement, and the diversity within Australian wild rice is of immense value for rice improvement and adaptation to environmental changes especially in the face of climate change. The reduction in variation in certain genomic regions of wild rice populations indicate bottlenecks induced by natural selection prior to domestication has also contributed to reduction in diversity in the rice genome (Figure 2). Biotic or abiotic stress in the environment of wild rice in tandem with reduced recombination [34] associated with the physical distribution of mutations [35] may explain loss of diversity in specific chromosome areas encoding genes contributing to adaptation to these environmental factors. This adaptation may have been important in the evolution of essential features of modern rice such as adaptation to an aquatic environment. Whole genome re-sequencing has enabled the identification of novel polymorphisms preserved in Australian A genome wild rices which would be useful in diversifying the ‘polymorphism deserts’ of cultivated rice [36].

## Supporting Information

File SI **This includes Figures S1 - S7, Tables S1 - S5 and Text S1.**
(DOCX)
