# Supplementary material for: Australian Wild Rice Reveals Pre-Domestication Origin of Polymorphism Deserts in Rice Genome
Source: PLoS One. 2014 Jun 6;9(6):e98843. doi: 10.1371/journal.pone.0098843 (PMC4048307; doi:10.1371/journal.pone.0098843)
Supplement: File SI — This includes Figures S1 - S7, Tables S1 - S5 and Text S1. (DOCX) [file pone.0098843.s001.docx]

**Supporting Information**

Supporting Texts: Text S1 – Text S3

Supporting Figures: Figure S1 – Text S7

Supporting Tables: Table S1 – Table S4

**Supporting Texts**

**Text S1.** Analysis of coverage in Chromosome 5 and in the polymorphism desert.

**Text S2.** Analysis of SNPs including SNPs from repetitive regions within the polymorphism desert.

**Text S3.** Analysis of SNPs variations in the regions associated with the domestication genes/ plant improvement genes in rice.

**Text S1. Analysis of coverage in Chromosome 5 and within the polymorphism desert**

A concern in the use of next generation sequencing platform is the extent and uniformity of coverage across the genome which have been sequenced. Therefore, in order to make sure that the lower number of polymorphisms observed in the ‘polymorphism desert’ of chromosome 5 is not due to less coverage in this region, the aligned data was subjected to an analysis which showed that the coverage across chromosome 5 was more than 4 reads in most of the regions (Figure S2).

Further, a fine analysis on the coverage across the ‘polymorphism desert’ was also carried out which also proved that the significant reduction in SNPs in the ‘polymorphism desert’ is not due to low coverage in the region (Figure S3).

**Text S2. Analysis of SNPs including SNPs from repetitive regions within the polymorphism desert.**

In the present study, only the SNPs from non-repetitive regions of the genome have been reported. Taking into consideration that the ‘polymorphism desert’ in Chromosome 5 is in the vicinity of the centromere, an analysis was carried out so as to ensure that eliminating the SNPs in repetitive did not have any effect on the SNP distribution in the ‘polymorphism desert’. SNPs distribution including SNPs from repetitive sequences reveal the reduction in SNPs is not due to filtering of SNPs from repetitive region (Figure S4).

**Text S3. Selection sweeps in the regions associated with the domestication genes/ plant improvement genes in rice.**

The analysis of SNP variation in the 2 Mb region centred on the 15 genes in rice which have been subjected to artificial selection either during domestication/ plant improvement namely namely *Gn1a* [7], *Rd* [8], *qSH1* [9], *sd1* [10] – [12] in chromosome 1, *GW2* [13] in chromosome 2, *GS3* [14] in chromosome 3, *GIF1* [15], *Bh4* [16], *sh4* [17] in chromosome 4, *qSW5* [18] in chromosome 5, *wx* [19] in chromosome 6, *PROG1* [20], *Rc* [21], *GBSSII* [22] in chromosome 7 and *BAD2* [23] in Chromosome 8 was carried out by analysing the SNPs variation in a sliding window of 1kb (Figure S6). The extent of sweeps due to selection was calculated by observing for regions with significant reduction in SNPs contiguous with the genes compared to the SNPs across the whole chromosome. Additionally, the assessment of coverage across the same 2 Mb region was assessed to calculate the exact sweeps eliminating the regions with low coverage (Figure S7).

In order to ascertain the exact extent of selection sweep in the seven genes, a fine analysis of the coverage in the 2 Mb region was carried out and the regions showing contiguity with the genes and sufficient coverage was marked out as the regions of low polymorphism due to the selection for the target genes in the cultivated rice (Figure S5).

1. Ashikari M, Sakakibara H, Lin S, Yamamoto T, Takashi T, et al. (2005) Cytokinin oxidase regulates rice grain production. Science 309: 741-745. doi: 10.1126/science.1113373.
2. Furukawa T, Maekawa M, Oki T, Suda I, Lida S, et al. (2007) The Rc and Rd genes are involved in proanthocyanidin synthesis in rice pericarp. Plant J 49: 91-102. doi: 10.1111/j.1365-313X.2006.02958.x.
3. Konishi S, Izawa T, Lin SY, Ebana K, Fukuta Y, et al. (2006) An SNP caused loss of seed shattering during rice domestication. Science 312: 1392-1396. doi: 10.1126/science.1126410.
4. Sasaki A, Ashikari M, Ueguchi-Tanaka M, Itoh H, Nishimura A, et al. (2002) Green revolution: a mutant gibberellin-synthesis gene in rice. Nature 416: 701–702. doi:10.1038/416701a.
5. Monna L, Kitazawa N, Yoshino R, Suzuki J, Masuda H, et al. (2002) Positional cloning of rice semidwarfing gene. sd-1: rice ‘green revolution gene’ encodes a mutant enzyme involved in gibberellin synthesis. DNA Res 9: 11–17. doi: 10.1093/dnares/9.1.11.
6. Spielmeyer W, Ellis MH, Chandler PM (2002) Semidwarf (sd-1), green revolution rice, contains a defective gibberellin 20-oxidase gene. Proc. Natl. Acad. Sci. USA 99: 9043–9048. doi: 10.1073/pnas.132266399.
7. Shomura A, Izawa T, Ebana K, Ebitani T, Kanegae H, et al. (2008) Deletion in a gene associated with grain size increased yields during rice domestication. Nat Genet 40: 1023-1028. doi:10.1038/ng.169.
8. Fan C, Xing Y, Mao H, Lu T, Han B, et al. (2006) *GS3*, a major QTL for grain length and weight and minor QTL for grain width and thickness in rice, encodes a putative transmembrane protein. Theor Appl Genet 112: 1164-1171. Doi: 10.1007/s00122-006-0218-1.
9. Wang E, Wang J, Zhu X, Hao W, Wang L, et al. (2008) Control of rice grain-filling and yield by a gene with a potential signature of domestication. Nat Genet 40: 1370–1374. doi: 10.1038/ng.220.
10. Zhu BF, Si L, Wang Z, Zhou Y, Zhu J, et al. (2011) Genetic control of a transition from black to straw-white seed hull in rice domestication. Plant Physiol 155: 1301–1311. doi: 10.1104/pp.110.168500.
11. Li C, Zhou A, Sang T (2006) Rice domestication by reducing shattering. Science 311: 1936-1939. doi: 10.1126/science.1123604.
12. Song XJ, Huang W, Shi M, Zhu MZ, et al. (2007) A QTL for rice grain width and weight encodes a previously unknown RING-type E3 ubiquitin ligase. Nat Genet 39: 623–630. doi:10.1038/ng2014.
13. Wang ZY, Zheng FQ, Shen GZ, Gao JP, Snustad DP, et al. (1995) The amylose content in rice endosperm is related to the post-transcriptional regulation of the *waxy* gene. Plant J.7, 613–622. doi: 10.1046/j.1365-313X.1995.7040613.x.
14. Tan L, Li X, Liu F, Sun X, Li C, et al. (2008) Control of a key transition from prostrate to erect growth in rice domestication. Nat Genet 40: 1360-1364. doi: 10.1038/ng.
15. Sweeney MT, Thomson MJ, Pfeil BE, McCouch S. (2006) Caught red-handed: *Rc* encodes a basic helix-loop-helix protein conditioning red pericarp in rice. Plant Cell 18: 283–294. doi: http:/​/​dx.​doi.​org/​10.​1105/​tpc.​105.​
16. Hirose T, Terao T (2004) A comprehensive expression analysis of the starch synthase gene family in rice (*Oryza sativa* L.). Planta 220(1): 9-16. doi: 10.1007/s00425-004-1314-6.
17. Bradbury LMT, Fitzgerald TL, Henry RJ, Jin Q, Waters DL (2005) The gene for fragrance in rice. Plant Biotechnol J 3: 363–370. doi: 10.1111/j.1467-7652.2005.00131.x.

**Supporting Figures**

**Figure S1.** Chromosome 5 SNP distribution based on *O. sativa* ssp. *indica* (cv. 93-11) reference genome.

**Figure S2.** Coverage across Chromosome 5.

**Figure S3.** Coverage and SNPs across the ‘polymorphism desert’ in Chromosome 5.

**Figure S4.** SNPs distribution across chromosome 5 including SNPs from repeat regions.

**Figure S5.** SNP distribution in the 2 Mb region centred on the genes involved in domestication/ plant improvement in rice.

**Figure S6.** Coverage in the 2 Mb region centred on the genes involved in domestication/ plant improvement in rice.

**Figure S7.** Extended regions of low polymorphism around 7 selected rice genes.

**
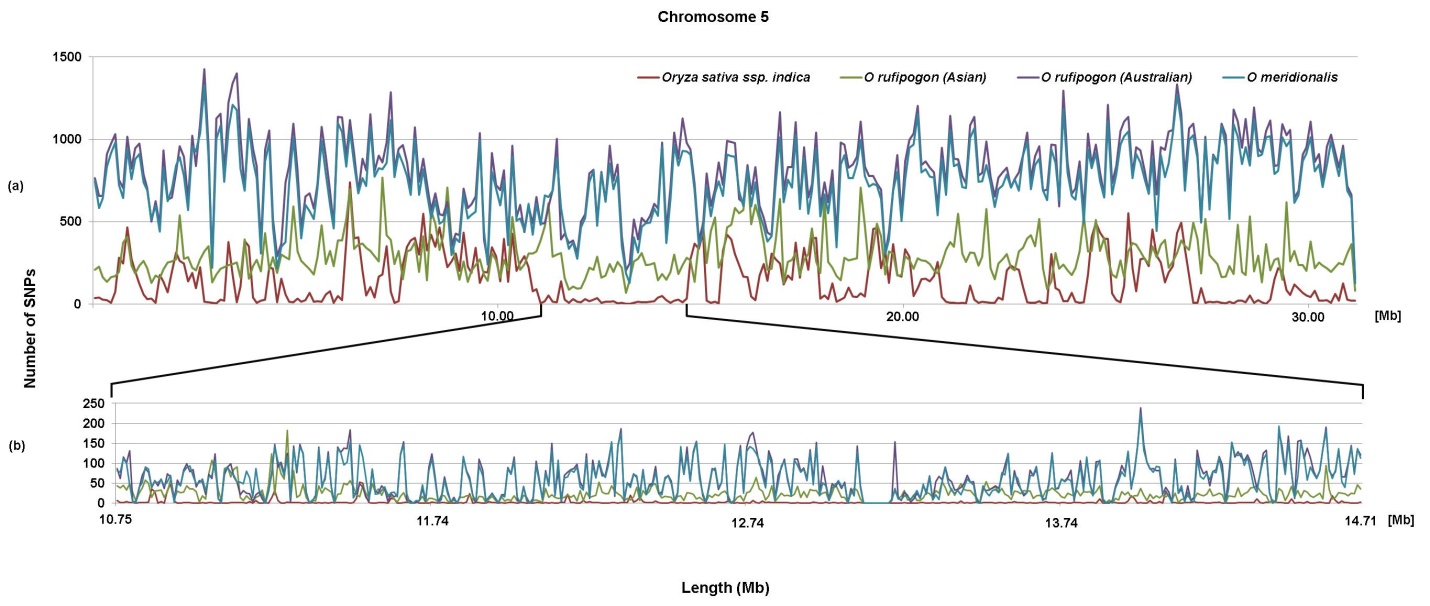
 Figure S1. Chromosome 5 SNP distribution based on *O. sativa* ssp. *indica* (cv. 93-11) reference genome.**

**
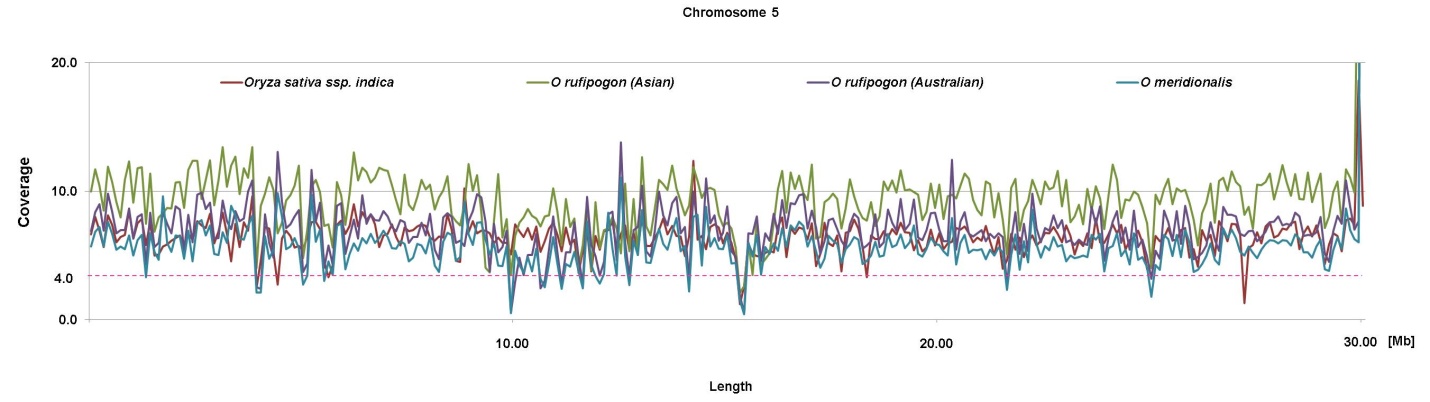
**

**Figure S2. Coverage across Chromosome 5.**


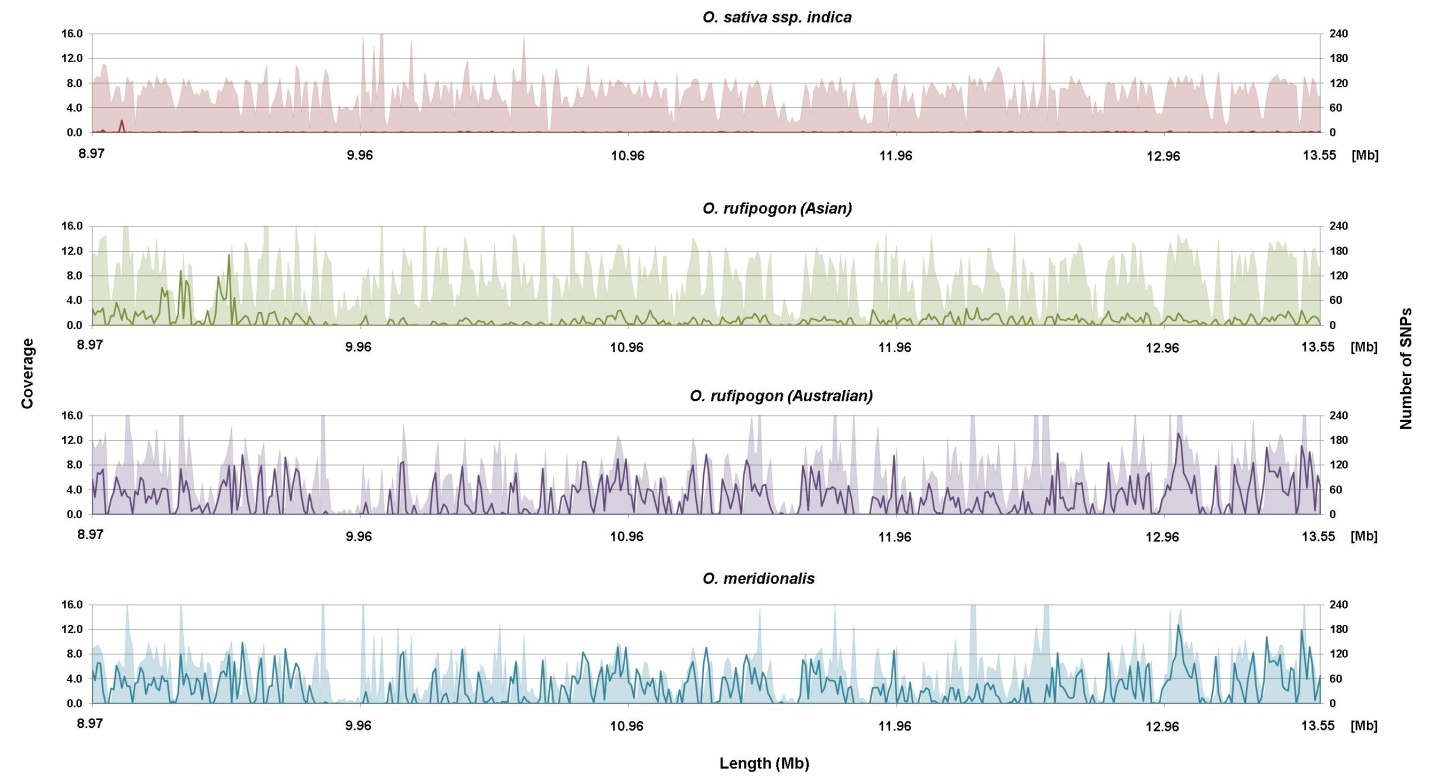


**Figure S3. Coverage and SNPs across the ‘polymorphism desert’ in Chromosome 5**; the shaded graph (in the primary axis) represents the coverage based on reads mapped to the reference genome while the line graph (in the secondary axis) shows the number of SNPs (excluding SNPs in repetitive regions) in the corresponding region.


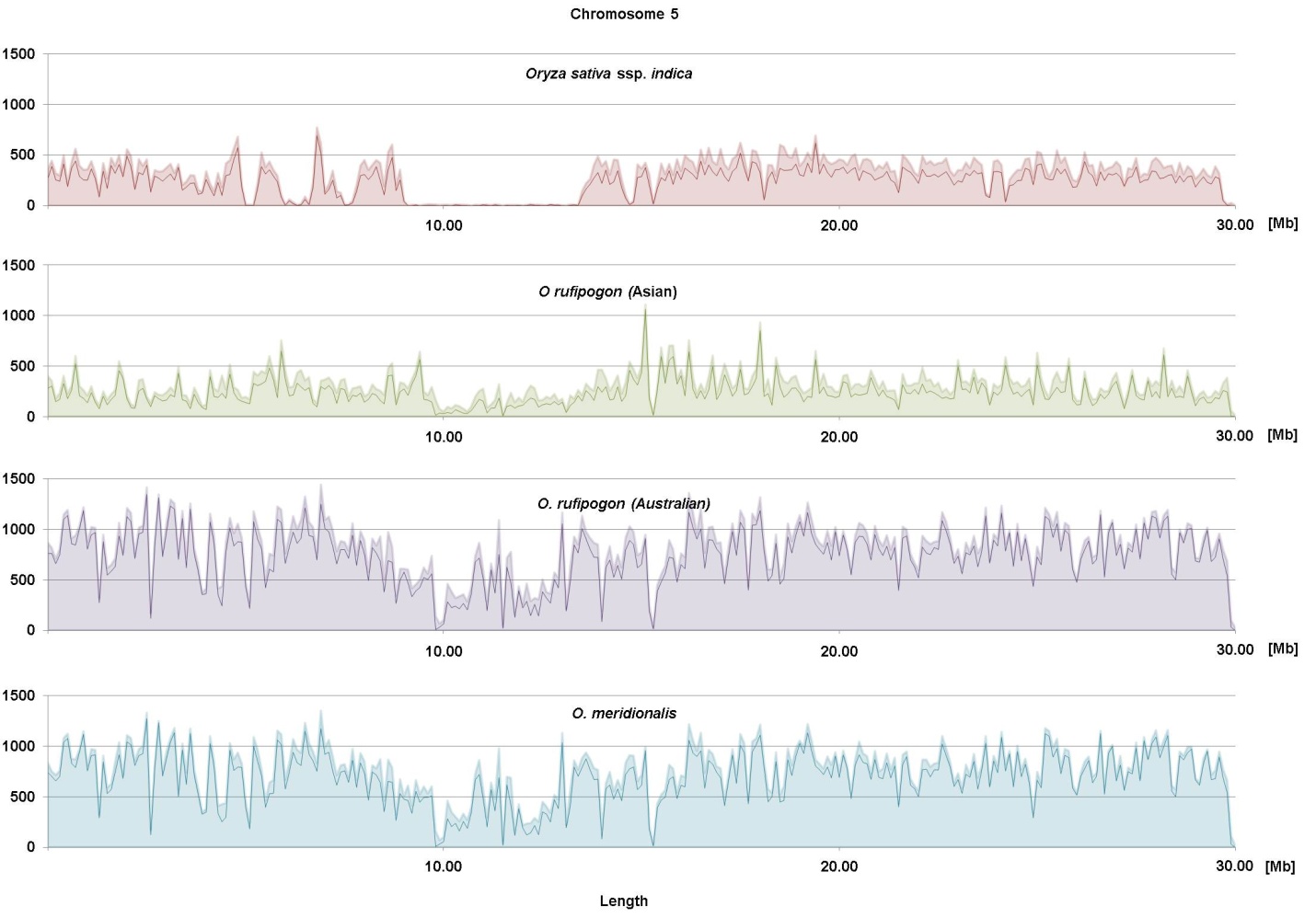
**Figure S4. SNPs distribution across chromosome 5 including SNPs from repeat regions**; the shaded graph represents the total number of SNPs (including SNPs from repetitive regions) while the line graph shows the number of SNPs excluding SNPs in reads mapped to repetitive regions.


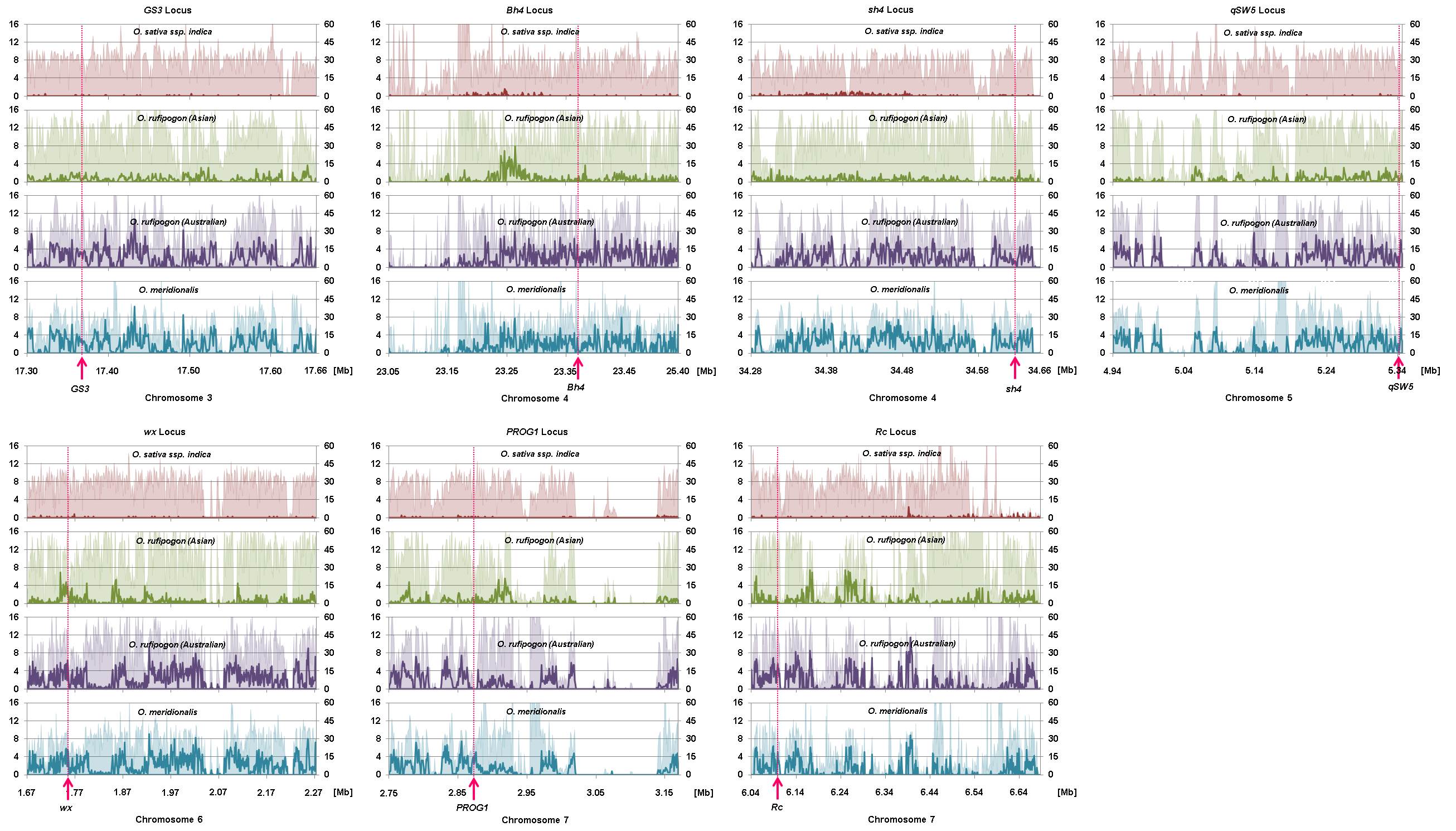


**Figure S5. Extended regions of low polymorphism around 7 selected rice genes**; the shaded graph (in the primary axis) represents the coverage based on reads mapped to the reference genome while the line graph (in the secondary axis) shows the number of SNPs (excluding SNPs in repetitive regions) in the corresponding region. The regions marked in red shades are regions without sufficient coverage and only the regions with sufficient coverage was considered for ascertaining the extent of selection sweep.


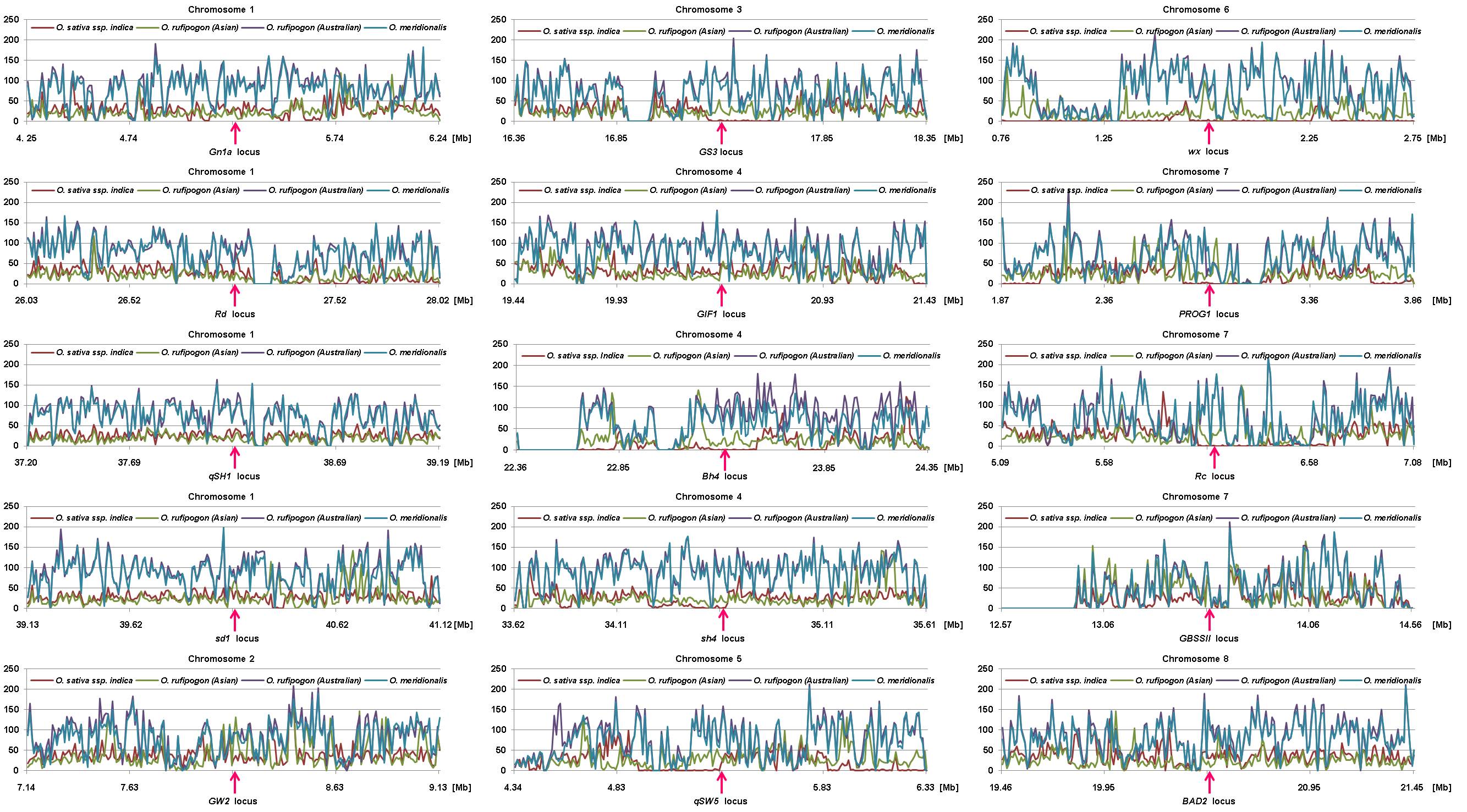


**Figure S6. SNP distribution in the 2 Mb region centred on the genes involved in domestication/ plant improvement in rice**; the genes which are highlighted in pink are the genes where there is significant reduction of SNPs which is associated with the selection pressure on these genes.


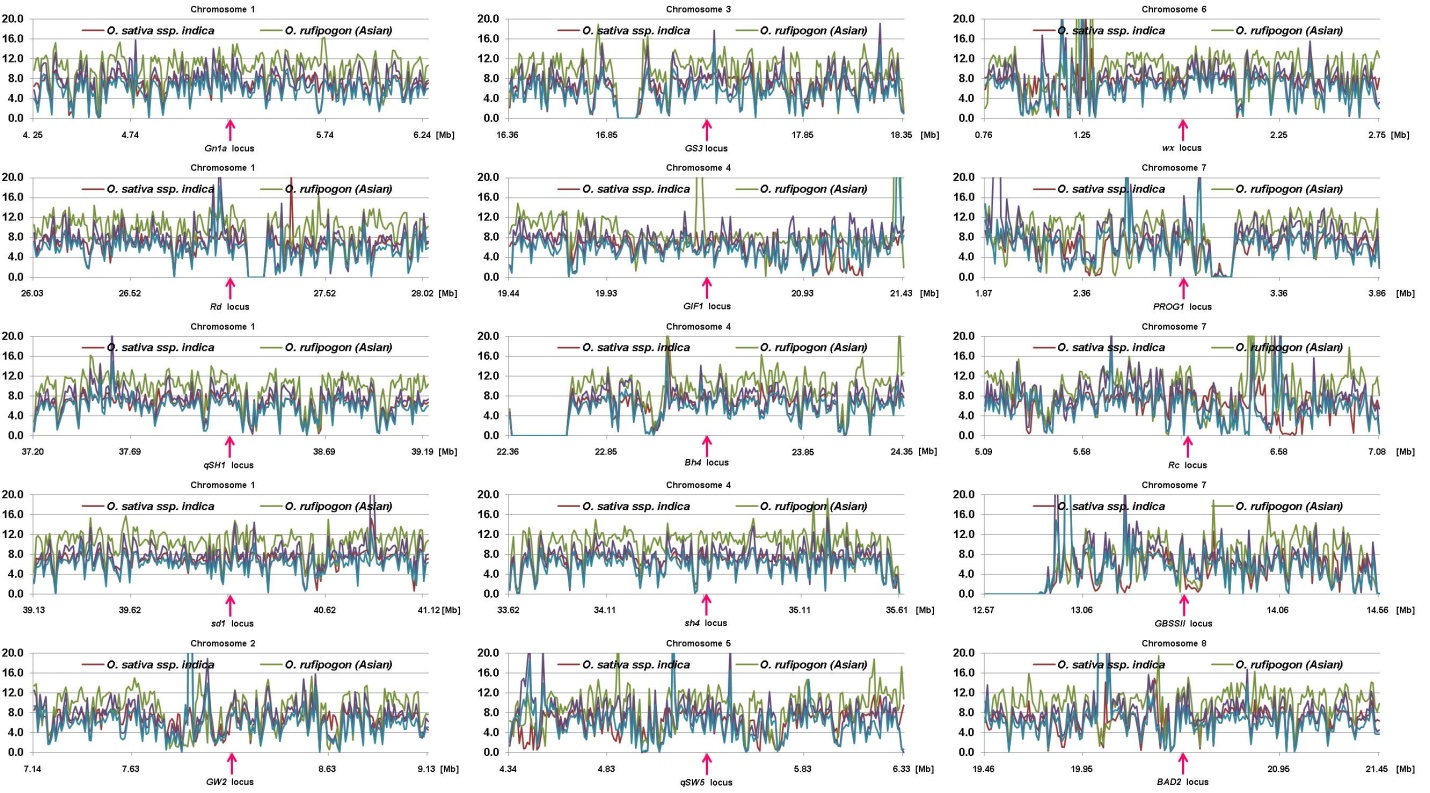


**Figure S7. Coverage in the 2 Mb region centred on the genes involved in domestication/ plant improvement in rice.**

**Supporting Tables**

**Table S1.** Genome-wide SNPs detected in the *Oryza* species in comparison to the Nipponbare reference genome.

**Table S2.** SNPs in genes of cultivated and wild *Oryza* as compared with Nipponbare genome.

**Table S3.** Non synonymous SNPs in genes of cultivated and wild *Oryza* as compared with Nipponbare genome.

**Table S4.** Mean SNPs per kb in 1 Mb genomic regions on either side of the rice domestication genes.

**Table S5.** Genes with low SNP rate in the Australian wild *Oryza* as compared with Nipponbare genome.

**Table S1. Genome-wide SNPs detected in the *Oryza* species in comparison to the Nipponbare reference genome.**

| ***Chromosome number*** | ***O. sativa* ssp. *indica*** | ***O. rufipogon* (Asia)** | ***O. rufipogon* (Australia)** | ***O. meridionalis*** |
| --- | --- | --- | --- | --- |
| Chromosome 1 | 123,276 (2.74) | 111,520 (2.47) | 325,074 (7.21) | 307,744 (6.83) |
| Chromosome 2 | 97,937 (2.66) | 92,064 (2.50) | 287,877 (7.82) | 271,312 (7.37) |
| Chromosome 3 | 94,548 (2.54) | 90,352 (2.43) | 293,657 (7.88) | 279,251 (7.50) |
| Chromosome 4 | 79,295 (2.21) | 85,801 (2.39) | 221,077 (6.16) | 209,857 (5.85) |
| Chromosome 5 | 68,443 (2.28) | 70,367 (2.34) | 219,794 (6.71) | 206,884 (6.89) |
| Chromosome 6 | 75,447 (2.35) | 81,084 (2.52) | 221,713 (6.90) | 207,937 (6.47) |
| Chromosome 7 | 78,275 (2.58) | 77,411 (2.55) | 198,138 (6.53) | 188,887 (6.22) |
| Chromosome 8 | 70,746 (2.48) | 65,678 (2.30) | 185,615 (6.51) | 175,594 (6.15) |
| Chromosome 9 | 61,722 (2.59) | 53,247 (2.24) | 153,855 (6.45) | 144,287 (6.05) |
| Chromosome 10 | 68,865 (2.91) | 56,362 (2.38) | 145,479 (6.15) | 138,738 (5.86) |
| Chromosome 11 | 82,494 (2.97) | 65,158 (2.35) | 151,672 (5.46) | 142,768 (5.14) |
| Chromosome 12 | 77,582 (2.52) | 68,894 (2.23) | 160,062 (5.19) | 144,825 (4.7) |
| Whole genome | 978,630 (2.5) | 917,738 (2.4) | 2,564,013 (6.7) | 2,418,084 (6.3) |

*Numbers in parenthesis represents the mean SNPs/ kb detected.

**Table S2. SNPs in genes of cultivated and wild *Oryza* as compared with Nipponbare genome.**

| ***Oryza* species** | **Whole genome (30,294 genes)** | | |  | **Chromosome 5 (1046 genes*)** | | |  | **Low Diversity Region of Chromosome 5 (93 genes**)** | | |
| --- | --- | --- | --- | --- | --- | --- | --- | --- | --- | --- | --- |
|  | **Total** | **SNPs/ gene** | **SNPs/kb of gene** |  | **Total** | **SNPs/ gene** | **SNPs/kb of gene** |  | **Total** | **SNPs/ gene** | **SNPs/kb of gene** |
| *O. sativa* ssp.*indica* | 229,507 | 7.58 | 2.12 |  | 8,113 | 7.76 | 2.27 |  | 26 | 0.28 | 0.05 |
| *O. rufipogon* (Asian) | 225,587 | 7.58 | 2.13 |  | 8,602 | 8.22 | 2.29 |  | 1136 | 12.22 | 2.12 |
| *O. rufipogon* (Australian) | 900,423 | 29.72 | 8.06 |  | 43,398 | 41.49 | 11.23 |  | 4479 | 48.16 | 9.47 |
| *O. meridionalis* | 867,051 | 28.62 | 7.84 |  | 41,889 | 40.05 | 10.81 |  | 4412 | 47.44 | 9.40 |

* Out of the 2398 genes in chromosomes 5 of IRGSP Pseudomolecules 4, only 1046 genes had uniform coverage of atleast 4 reads across all the four genomes sequenced.

* Out of the 143 genes in the ‘polymorphism desert’ of chromosomes 5 of IRGSP Pseudomolecules 4, only 93 genes had uniform coverage of atleast 4 reads across all the four genomes sequenced.

**Table S3. Non synonymous SNPs in genes of cultivated and wild *Oryza* as compared with Nipponbare genome.**

| ***Oryza* species** | **Whole genome (30,294 genes)** | | |  | **Chromosome 5 (1046 genes*)** | | |  | **Low Diversity Region of Chromosome 5 (93 genes**)** | | |
| --- | --- | --- | --- | --- | --- | --- | --- | --- | --- | --- | --- |
|  | **Total** | **nsSNPs/ gene** | **nsSNPs/kb of gene** |  | **Total** | **nsSNPs/ gene** | **nsSNPs/kb of gene** |  | **Total** | **nsSNPs/ gene** | **nsSNPs/kb of gene** |
| *O. sativa*ssp.*indica* | 27,672 | 0.91 | 0.30 |  | 929 | 0.89 | 0.31 |  | 4 | 0.04 | 0.01 |
| *O. rufipogon* (Asian) | 26,581 | 0.88 | 0.31 |  | 937 | 0.90 | 0.28 |  | 105 | 1.13 | 0.22 |
| *O. rufipogon* (Australian) | 82,270 | 2.72 | 0.89 |  | 3643 | 3.48 | 1.09 |  | 351 | 3.77 | 3.94 |
| *O. meridionalis* | 78,839 | 2.60 | 0.87 |  | 3636 | 3.48 | 1.12 |  | 366 | 0.98 | 1.04 |

* Out of the 2398 genes in chromosomes 5 of IRGSP Pseudomolecules 4, only 1046 genes had uniform coverage of atleast 4 reads across all the four genomes sequenced.

* Out of the 143 genes in the ‘polymorphism desert’ of chromosomes 5 of IRGSP Pseudomolecules 4, only 93 genes had uniform coverage of atleast 4 reads across all the four genomes sequenced.

**Table S4. Mean SNPs per kb in 1 Mb genomic regions on either side of the rice domestication genes.**

| ***Chromosome/ Locus*** | ***O. sativa* ssp. *indica*** | ***O. rufipogon* (Asia)** | ***O. rufipogon* (Australia)** | ***O. meridionalis*** |
| --- | --- | --- | --- | --- |
| **Chromosome 1** | **2.74 (123,276)** | **2.47 (111,520)** | **7.21 (325,074)** | **6.83 (307,744)** |
| *Gn1a* | 2.79 (5,591) | 2.38 (4,777) | 8.31 (16,684) | 7.79 (15,626) |
| *Rd* | 2.28 (4,567) | 2.02 (4,040) | 7.42 (14,868) | 7.19 (14,404) |
| *qSH1* | 2.42 (4,862) | 1.79 (3,588) | 7.48 (14,990) | 7.09 (14,207) |
| *sd1* | 2.83 (5,673) | 2.80 (5,621) | 8.92 (17,868) | 8.37 (16,783) |
| **Chromosome 2** | **2.66 (97,937)** | **2.50 (92,064)** | **7.82 (287,877)** | **7.37 (271,312)** |
| *GW2* | 3.85 (7,740) | 3.99 (8,007) | 8.26 (16,583) | 7.71 (15,491) |
| **Chromosome 3** | **2.54 (94,548)** | **2.43 (90,352)** | **7.88 (293,657)** | **7.50 (279,251)** |
| *GS3* | 2.47 (4,964) | 2.46 (4,940) | 7.09 (14,238) | 6.59 (13,224) |
| **Chromosome 4** | **2.21 (79,295)** | **2.39 (85,801)** | **6.16 (221,077)** | **5.85 (209,857)** |
| *GIF1* | 3.18 (6,358) | 2.83 (5670) | 8.59 (17,194) | 8.06 (16,126) |
| *Bh4* | 1.27 (2,550) | 1.92 (3,843) | 6.67 (13,359) | 5.24 (10,498) |
| *sh4* | 2.42 (4,841) | 2.38 (4,772) | 7.31 (14,667) | 8.86 (17,763) |
| **Chromosome 5** | **2.28 (68,443)** | **2.34 (70,367)** | **6.71 (219,794)** | **6.89 (206,884)** |
| *qSW5* | 1.98 (3,960) | 2.86 (5,722) | 7.40 (14,801) | 6.64 (13,294) |
| Polymorphism desert | 0.04 (196) | 1.32 (6,050) | 3.96 (18,176) | 3.74 (17,139) |
| **Chromosome 6** | **2.35 (75,447)** | **2.52 (81,084)** | **6.90 (221,713)** | **6.47 (207,937)** |
| *wx* | 0.29 (588) | 2.11 (4,242) | 8.06 (16,184) | 7.61 (15,270) |
| **Chromosome 7** | **2.58 (78,275)** | **2.55 (77,411)** | **6.53 (198,138)** | **6.22 (188,887)** |
| *PROG1* | 1.86 (3,714) | 2.69 (5,389) | 6.81 (13,628) | 6.49 (12,990) |
| *Rc* | 2.57 (5,163) | 2.59 (5,194) | 6.85 (13,745) | 5.03 (10,095) |
| *GBSSII* | 2.20 (4,416) | 3.09 (6,216) | 4.78 (9,609) | 4.61 (9,257) |
| **Chromosome 8** | **2.48 (70,746)** | **2.30 (65,678)** | **6.51 (185,615)** | **6.15 (175,594)** |
| *BAD2* | 3.55 (7,121) | 2.47 (4,962) | 8.31 (16,684) | 7.80 (15,660) |
| **Whole genome** | **2.56 (978,630)** | **2.40 (917,738)** | **6.71 (2,564,013)** | **6.33 (2,418,084)** |

*Numbers in parenthesis is the total number of SNPs detected in the respective regions in comparison with Nipponbare genome.

**Table S5. Genes with low SNP rate in the Australian wild *Oryza* as compared with Nipponbare genome.**

| **Gene ID** | **Mean SNPs per kb** | | | | **Gene Functions** |
| --- | --- | --- | --- | --- | --- |
|  | ***O. sativa*ssp. *indica*** | ***O. rufipogon* (Asian)** | ***O. rufipogon* (Australian)** | ***O. meridionalis*** |  |
| Os05g0252000 | 0.0 | 8.7 | 4.5 | 5.5 | Aerobic seed germination; Inflorescence and seed development |
| Os05g0255800 | 0.3 | 6.1 | 4.7 | 6.1 | Inflorescence and seed development; BB infection |
| Os05g0256100 | 0.0 | 14.9 | 7.5 | 7.3 | Aerobic germination |
| Os05g0258400 | 0.0 | 2.8 | 8.9 | 8.8 | Cytokinin response in roots&leaves; gibberellin signalling; Fe&P interaction |
| Os05g0267900 | 0.0 | 0.9 | 7.1 | 7.7 | Gibberellin signalling |
| Os05g0268500 | 0.0 | 0.8 | 5.3 | 5.2 | Inflorescence and seed development |
| Os05g0272800 | 0.0 | 2.3 | 9.8 | 7.5 | Aerobic germination; BB infection |
| Os05g0274200 | 0.0 | 0.3 | 8.6 | 8.3 | Inflorescence& seed development |
| Os05g0276500 | 0.0 | 2.2 | 9.0 | 6.2 | Aerobic germination; Fe&P interaction: cytokinin response in roots |
| Os05g0280200* | 0.0 | 0.0 | 4.9 | 4.3 | Aerobic germination |
| Os05g0280500 | 0.0 | 1.3 | 9.0 | 9.0 | Aerobic germination; Fe&P interaction: cytokinin response in roots |
| Os05g0280700* | 0.3 | 0.3 | 3.5 | 3.2 | Leaf&stem photo thermoperiod; Fe&P interaction |
| Os05g0291700* | 0.0 | 1.4 | 4.8 | 4.8 | Cytokinin response in roots&leaves; inflorescence and seed development; Fe&P interaction |
| Os05g0297900 | 0.0 | 0.3 | 8.3 | 8.0 | Rice stripe virus infection |
| Os05g0298200 | 0.0 | 1.2 | 9.5 | 10.0 | BB infection |
| Os05g0299100 | 0.0 | 2.3 | 7.6 | 8.4 | Fe& P interaction |

*Genes with significantly lower SNPs and ns SNPs per kb in *O. rufipogon* and *O. meridionalis* from Australia as well.
